# Adelmidrol, in combination with hyaluronic acid, displays increased anti-inflammatory and analgesic effects against monosodium iodoacetate-induced osteoarthritis in rats

**DOI:** 10.1186/s13075-016-1189-5

**Published:** 2016-12-12

**Authors:** Rosanna Di Paola, Roberta Fusco, Daniela Impellizzeri, Marika Cordaro, Domenico Britti, Valeria Maria Morittu, Maurizio Evangelista, Salvatore Cuzzocrea

**Affiliations:** 1 Department of Chemical, Biological, Pharmaceutical and Environmental Sciences, University of Messina, Viale Ferdinando Stagno D’Alcontres, n 31, Messina, 98166 Italy; 2 Department of Health Science, University of Catanzaro, Viale Europa, Campus S. Venuta, Germaneto, Catanzaro, 88100 Italy; 3 Institute of Anaesthesiology and Reanimation, Catholic University of the Sacred Heart, Rome, Italy; 4 Department of Pharmacological and Physiological Science, Saint Louis University School of Medicine, 1402 South Grand Blvd, St. Louis, MO 63104 USA

**Keywords:** Osteoarthritis, Adelmidrol, Sodium hyaluronate, Inflammation, Cartilage degeneration

## Abstract

**Background:**

Osteoarthritis (OA) is a degenerative joint disease produced by a cascade of events that can ultimately lead to joint damage. The aim of this study was to evaluate the effect of adelmidrol, a synthetic palmitoylethanolamide analogue, combined with hyaluronic acid on pain severity and modulation of the inflammatory response in a rat model of monosodium iodoacetate (MIA)-induced osteoarthritis.

**Methods:**

OA was induced by intra-articular injection of MIA in the knee joint. On day 21 post-MIA administration, the knee joint was analyzed. Rats subjected to OA were treated by intra-articular injection of adelmidrol in combination with sodium hyaluronate at different doses and time points after MIA induction. Limb nociception was assessed by the paw withdrawal latency and threshold measurement. Samples were examined macroscopically, histologically, and by immunohistochemistry.

**Results:**

At day 21 post-MIA injection, the MIA + solvent and MIA + 1.0% sodium hyaluronate groups showed irregularities and fibrillation in the surface layer, a decrease in blood cells and multilayering in transition and radial zones, no pannus formation, and modified Mankin scores significantly higher than sham knees. The combination of hyaluronic acid and adelmidrol dose-dependently (adelmidrol 0.6% + 1.0% sodium hyaluronate and adelmidrol 2% + 1.0% sodium hyaluronate) reduced the histological alterations induced by MIA. Moreover, degeneration of articular cartilage, mast cell infiltration, and pro-inflammatory cytokine and chemokine plasma levels were significantly downregulated by treatment with a combination of hyaluronic acid and adelmidrol at the above doses.

**Conclusions:**

Our results clearly demonstrate that the combination of hyaluronic acid and adelmidrol improves the signs of OA induced by MIA.

## Background

Osteoarthritis (OA) is one of the most common joint disabling disorders in adults [1]. It occurs when the protective cartilage on the ends of bones breaks down, causing pain, swelling, and problems in joint articulation [2]. OA is primarily characterized by degeneration of cartilage at the joints but also implicates other pathological changes, including synovial inflammation, osteophyte formation, and subchondral bone sclerosis, in all tissues of joints [3–5]. OA can affect any joint, but the disorder most frequently affects joints in the hands, knees, hips, and spine, where it induces stiffness and joint dysfunction. Current guidelines defined by The Osteoarthritis Research Society International state that OA treatment should be aimed at reducing pain and joint stiffness, maintaining and improving joint mobility, reducing physical disability, and improving patient quality of life by limiting progression of damage [6].

The monosodium iodoacetate (MIA) experimental model is commonly used as an animal model of arthritis pain associated with OA [7]. In particular, the MIA model has been widely used for the pharmacological evaluation of new drug therapy [8]. Nonsteroidal anti-inflammatory drugs (NSAIDs) and steroids reduce the OA symptoms of joint pain and swelling [9]. However, evidence in clinics shows that pharmacological interventions, including acetaminophen, NSAIDS, topical agents, and intra-articular injections (e.g., steroids), and non-pharmacological interventions, such as joint replacement, are both sparse and controversial [10–12]. Glucocorticoids (GCs) have powerful immunosuppressive effects and are widely used in the management of chronic inflammatory diseases. Long-term therapy with GCs is often necessary to control the symptoms of osteoarthritis [13]. Important evidence shows that GCs are also used in association with hyaluronic acid (HA) and partially show an ability to reduce pain as well as the progression of disease [14]. A number of studies suggest that HA associated with GCs might have a beneficial effect partially related to a viscoelastic lubricant effect [15, 16].

A major drawback in the use of HA relates to the ability of the intra-articular environment to depolymerize the polysaccharide with a speed directly proportional to the degree of intra-articular inflammation. The correct degradation of HA is therefore essential to maintain the integrity of tissues and, in particular, joint homeostasis. Moreover, an important question mark remains about therapeutic management of long-term pathologies, with steroids often linked to a series of unwanted side effects [17].

Adelmidrol is a semisynthetic derivative of azelaic acid and analogue of the anti-inflammatory compound palmitoylethanolamide (PEA), an aliamide and member of the family of fatty acid amide signaling molecules with cannabimimetic properties. The anti-inflammatory and antinociceptive effects of the aliamides PEA and adelmidrol have been demonstrated in numerous pre-clinical studies, both in vitro and in vivo [18–20]. Their actions are thought to be due, at least in part, to their ability to down-modulate mast cell activation and mast cell mediator release in pathophysiological and pathological conditions [21].

The aim of the present study was to demonstrate that adelmidrol in association with HA is able to control depolymerization of exogenous HA, by bringing about a viscoelastic-type lubricating action and consequent modulation of inflammatory processes and pain in a rat model of MIA-induced OA.

## Methods

### Animals

Forty male Lewis rats (Sprague-Dawley, 200–230 g; Harlan, Nossan Milan, Italy) were maintained in a monitored environment and provided with standard rodent chow and water. The study was authorized by the University of Messina Review Board for the care of animals (Protocol number 8/U-apr16). Animal care was in conformity with Italian regulations for the protection of experimental animals (DM 116192) and with European Economic Community regulations (OJ of EC L 358/1 12/18/1986).

### Experimental protocol

OA was induced by intra-articular injection of MIA in the knee joint [22]. On day 0, rats were anesthetized with 5.0% isoflurane (Baxter International). A volume of 25 μl sterile saline solution + 3 mg MIA was injected into the knee joint through the right infrapatellar ligament. The left knee received an equal volume of 0.9% sterile saline. MIA was prepared in sterile conditions and injected using a 50-μl Hamilton syringe with a 27-gauge needle that was inserted into the joint to about 2–3 mm. On day 21 post-MIA administration, knee joints were inspected in detail to determine histopathological changes. Cartilage was stained to verify the presence of OA or not.

### Experimental groups

Rats were randomly divided into the following groups:

#### MIA + vehicle *(solvent solution)*

Rats were subjected to induction of OA as described above, and received 25 μl of the solvent solution in the infrapatellar area of the right knee at days 3, 7, 14, and 21 (*n* = 10) by intra-articular injection.

#### MIA adelmidrol 0.6% + 1.0% *sodium hyaluronate*

Rats were subjected to induction of OA as described above, and were treated by intra-articular injection of adelmidrol 0.6% + sodium hyaluronate 1.0% (sodium hyaluronate with high molecular weight, between 1.5 and 2.0 million daltons) at a dose of 150 μg/25 μl on days 3, 7, 14, and 21 after MIA induction (*n* = 10)

#### MIA adelmidrol 2% + sodium hyaluronate 1.0%

Rats were subjected to induction of OA as described above, and were treated by intra-articular injection of adelmidrol 2% + sodium hyaluronate 1.0% (sodium hyaluronate with high molecular weight, between 1.5 and 2.0 million daltons) at a dose of 150 μg/25 μl on days 3, 7, 14, and 21 after MIA induction (*n* = 10)

#### MIA + sodium hyaluronate 1.0%

Rats were subjected to induction of OA as described above, and were treated by intra-articular injection of sodium hyaluronate 1.0% (sodium hyaluronate with high molecular weight, between 1.5 and 2.0 million daltons) at a dose of 150 μg/25 μl on days 3, 7, 14, and 21 after MIA induction (*n* = 10)

#### Sham group

Rats were administered by intra-articular injection with 0.9% saline (25 μl) instead of MIA and were treated with either vehicle or different formulations on days 3, 7, 14, and 21 (*n* = 10)

### Pain measurement

Mechanical sensitivity was evaluated using a dynamic plantar aesthesiometer (Ugo Basile, Comerio, Italy). The rats were placed on a metal mesh surface in a chamber in a room with a controlled temperature (22 °C) and they were allowed to adapt for 15 min before the testing began. The touch stimulator part was oriented under the animal. When the aesthesiometer was activated, a plastic monofilament touched the paw in the proximal metatarsal region. The filament exercised a gradually increasing force on the plantar, starting below the threshold of detection and increasing until the stimulus became painful and the rat removed its paw. The force required to produce a paw withdrawal reflex was recorded automatically and measured in grams. A maximum force of 50 g and a ramp speed of 20 s were used for all the aesthesiometry tests.

### Analysis of motor function (Walking Track Analysis)

The rat was placed in a walking track with a dark end. White office paper of the appropriate size was placed on the bottom of the track. Hind limbs of the rat were dipped in ink, and the rat was allowed to walk along the track, leaving paw prints on the paper. The test was performed before induction on day 0 and at days 3, 7, 14, and 21 after induction [23]. The functionality index of the sciatic nerve (SFI), calculated by Walking Track Analysis, was evaluated at 60 min after the injection on days 3, 7, 14, and 21: values close to 0 indicate normal functioning, and values tending to –100 indicate an alteration of sciatic nerve functionality [24].

### Micro-computed tomography analysis

In order to evaluate the bone mass and microarchitecture parameters, including the fraction of bone volume, the proximal and distal parts of the right tibiae were scanned using micro-computed tomography (Micro-CT; Skyscan, Belgium) The scan conditions were as follows: an aluminum filter of 0.5 mm, X-ray voltage of 50Kv, X-ray current of 200 mA, and an exposure time of 360 ms. After scanning, the cross-sectional slices were reconstructed and three-dimensional analyses were performed using CTAn SkyScan software.

### Histological analysis

On day 21 after MIA administration, rats were sacrificed by anesthetic overdose and perfused with 4% paraformaldehyde solution. Tibiofemoral joints were collected and post-fixed in neutral buffered formalin (containing 4% formaldehyde), decalcified in EDTA, and processed as following described. After decalcification, the specimens were embedded in paraffin. Mid-coronal tissue sections (5 μm) were stained for evaluation; all histomorphometric analyses were performed by an observer blinded to the treatment groups. Sections were stained with hematoxylin and eosin and observed by light microscopy (Dialux 22 Leitz; Leica Microsystems SpA, Milan, Italy). Histopathological analysis of the cartilage was assessed by the modified score of Mankin [14] (score range 0 to 12, from normal to complete disorganization and hypocellularity).

Cartilage degeneration was assessed by staining with toluidine blue and analyzed using the following criteria described by Janusz et al. [25]: 1 = mild into the surface region; 2 = slightly extended in the upper center; 3 = moderate in the median area; 4 = extended area deep; and 5 = severe degeneration.

### Mast cell staining

Identification of mast cells was performed as described previously [26]. Knee sections were cut at 5-μm thickness and stained with 0.25% toluidine blue, pH 2.5, for 45 min at room temperature. The sections were then dehydrated and mounted for viewing. Three non-sequential sections were chosen by a block at random from each paw for examination. All sections were evaluated at 200×, while some sections were photographed at 400× using a Nikon inverted microscope. The density of mast cells is expressed as the number of mast cells per unit area of bone tissue.

### Measurement of cytokines, metalloproteinases, and nerve growth factor

The levels of tumor necrosis factor (TNF)-α, interleukin (IL)-1β, nerve growth factor (NGF), and matrix metalloproteinase (MMP)-1, MMP-3, and MMP-9 were measured in serum. Assays were performed using commercial colorimetric enzyme-linked immunosorbent assay (ELISA) kits (TNF-α, IL-1β, and NGF: Thermo Fisher Scientic; MMP-1, MMP-3, and MMP-9: Cusabio).

### Reagents

Unless otherwise stated, all compounds were obtained from Sigma-Aldrich Co. All other chemicals were of the highest commercial grade available. All stock solutions were prepared in non-pyrogenic saline (0.9% NaCl; Baxter Healthcare Ltd., Thetford, Norfolk, UK).

### Data analysis

All values in the figures and text are expressed as mean ± standard error of the mean (SEM) of *n* observations. For in vivo studies, *n* represents the number of animals studied. In experiments involving histology, the figures shown are representative of at least three experiments performed on different days. The results were analyzed by one-way analysis of variance (ANOVA) followed by a Bonferroni post-hoc test for multiple comparisons. Non-parametric data were analyzed with the Fisher's exact test. A *P* value less than 0.05 is considered significant.

## Results

### Effects of the HA and adelmidrol combination on OA pain production and motor function

**Fig. 1:**
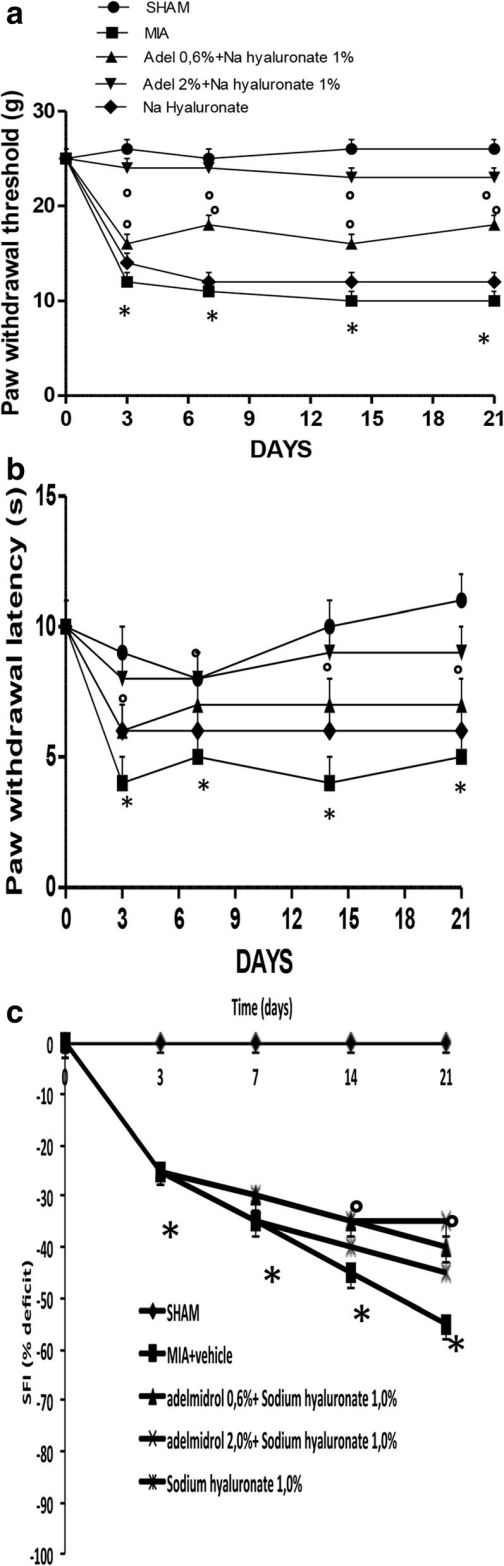
Effects of sodium hyaluronate (*Na hyaluronate*) and adelmidrol (*Adel.*) combination on OA pain production and motor function. In the von Frey hair assessment test, the paw withdrawal threshold (PWT) (**a**) and the paw withdrawal latency (PWL) (**b**) were prolonged significantly in the inflamed hind paw of the rats given 0.6% adelmidrol + 1.0% sodium hyaluronate and 2% adelmidrol + 1.0% sodium hyaluronate compared with the monosodium iodoacetate (*MIA*) + solvent group and MIA + sodium hyaluronate 1.0% group. The sham group showed no obvious change. **c** In the MIA + vehicle and 1.0% sodium hyaluronate groups, the sciatic nerve functionality index (*SFI*) values were significantly lower than in sham animals. Treatment with a combination of HA acid and adelmidrol improved motor function in a dose-dependent manner. Data are means ± S.E.M. of 10 rats for each group. *P < 0.05 versus sham, °*P* < 0.05 versus MIA + vehicle

Because pain is the predominant symptom of OA, the secondary tactile allodynia in MIA-induced OA rats was assessed. In the von Frey hair assessment test, the paw withdrawal latency (PWL) (Fig. 1b) and the paw withdrawal threshold (PWT) (Fig. 1a) were significantly prolonged in the inflamed hind paw of the rats given 0.6% adelmidrol + 1.0% sodium hyaluronate and 2% adelmidrol + 1.0% sodium hyaluronate compared with the MIA + solvent group and the MIA + sodium hyaluronate 1.0% group, demonstrating the antinociceptive property of adelmidrol in a dose-dependent manner. The sham group showed no obvious change (Fig. 1a and b).

Moreover, we tested motor function using a walking track in order to demonstrate that the association of adelmidrol with sodium hyaluronate treatment may improve joint mobility and reduce physical disability. Analysis was performed on days 3, 7, 14, and 21 after induction of OA. As shown in Fig. 1c, the SFI in the sham group was normal throughout the experiment, with SFI approximating 0. In the MIA + vehicle and 1.0% sodium hyaluronate groups, SFI values on post-MIA administration on days 3, 7, 14, and 21 were significantly lower than those observed in the sham group. The combination of HA and adelmidrol (0.6% adelmidrol + 1.0% sodium hyaluronate and 2% adelmidrol + 1.0% sodium hyaluronate) dose-dependently improved motor function (Fig. 1c).

### Effects of the HA and adelmidrol combination on plasma levels of pro-inflammatory cytokines and NGF

**Fig. 2:**
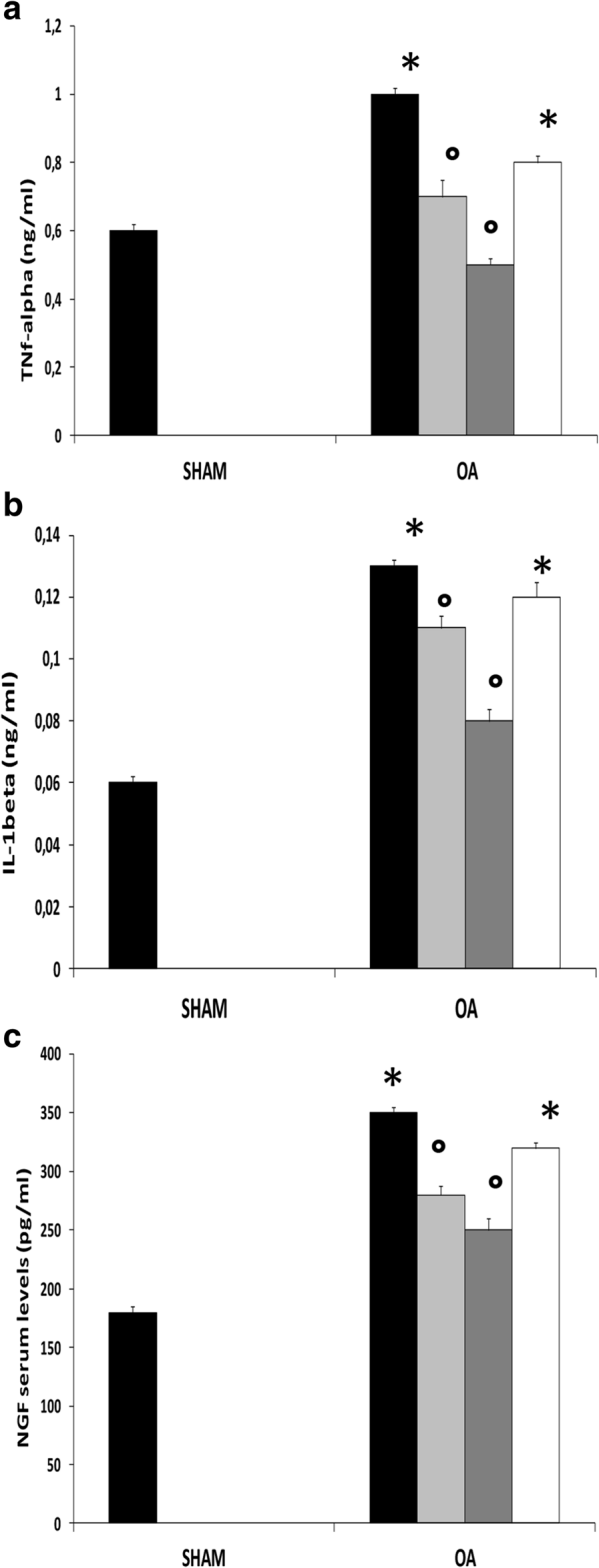
Effects of the combination of hyaluronic acid and adelmidrol on plasma cytokines and nerve growth factor (*NGF*) in osteoarthritis (*OA*) rats. MIA-induced OA and tissue sample processing were carried out as detailed in the Methods section. Increased tumor necrosis factor alpha (*TNF-α*) (**a**), interleukin-1beta (*IL-1β*) (**b**), and NGF (**c**) plasma levels were detected in the MIA + solvent and MIA + 1.0% sodium hyaluronate groups after MIA administration. OA rats treated with the indicated doses of adelmidrol and HA exhibited a dose-dependent reduction in the plasma levels of all the measured parameters. Values are shown as mean ± SEM of 10 animals for each group. *P < 0.05 versus sham, °*P* < 0.05 versus MIA + vehicle.

To test whether the association of adelmidrol with sodium hyaluronate treatment may modulate the secretion of pro-inflammatory cytokines, we analyzed plasma levels of TNF-α, IL-1β, and NGF. A significant increase in serum levels of TNF-α (Fig. 2a), IL-1β (Fig. 2b), and NGF (Fig. 2c) was observed in the MIA + vehicle and 1% sodium hyaluronate groups compared to sham animals. In contrast, the HA and adelmidrol combination (0.6% adelmidrol + 1.0% sodium hyaluronate and 2% adelmidrol + 1.0% sodium hyaluronate) dose-dependently reduced the increase in serum levels of TNF-α (Fig. 2a), IL-1β (Fig. 2b), and NGF (Fig. 2c) after administration of MIA.

### Effects of the HA and adelmidrol combination on mast cell infiltration

**Fig. 3:**
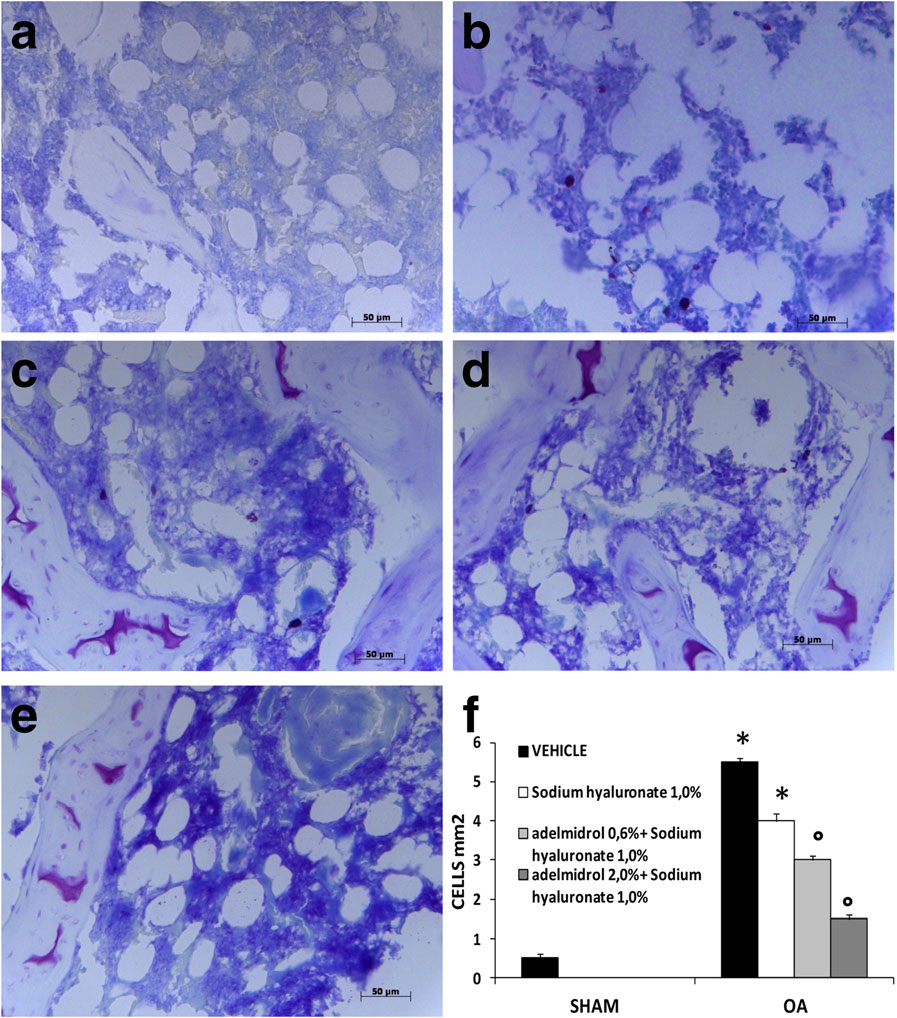
Effects of the combination of hyaluronic acid and adelmidrol on mast cell staining in osteoarthritis (*OA*) knee tissue. Compared with sham knees (**a**), the MIA + solvent (**b**) and MIA + 1.0% sodium hyaluronate (**c**) groups displayed significant increases in the numerical density of toluidine blue-positive cells. In contrast, the combination of HA and adelmidrol at the doses indicated (0.6% adelmidrol + 1.0% sodium hyaluronate (**d**), and 2% adelmidrol + 1.0% sodium hyaluronate (**e**)) produced a dose-dependent reduction in mast cell infiltration in the knees of MIA-treated rats. (**f**) Number of mast cells per unit area of muscle parenchyma (mast cell density). Data are means ± SEM of 10 rats for each group. *P < 0.05 versus sham, °*P* < 0.05 versus MIA + vehicle

An important source of cytokines, specifically TNF-α and IL-1β, is characterized by mast cell activation. Mast cell infiltration after OA induction was studied by staining knee joint tissues with toluidine blue. Degranulated mast cells were not seen in knee sections from the sham group (Fig. 3a; mast cell density in Fig. 3f). On the contrary, significant mast cell infiltration was observed in the MIA + solvent (Fig. 3b) and MIA + 1.0% sodium hyaluronate (Fig. 3c) groups in joint tissue near the subchondral bone taken 21 days after the induction of OA. The combination of HA and adelmidrol (0.6% adelmidrol + 1.0% sodium hyaluronate and 2% adelmidrol + 1.0% sodium hyaluronate) (Fig. 3d and e) dose-dependently reduced mast cell infiltration induced by administration of MIA.

### Effects of the HA and adelmidrol combination on plasma levels of metalloproteinases

**Fig. 4:**
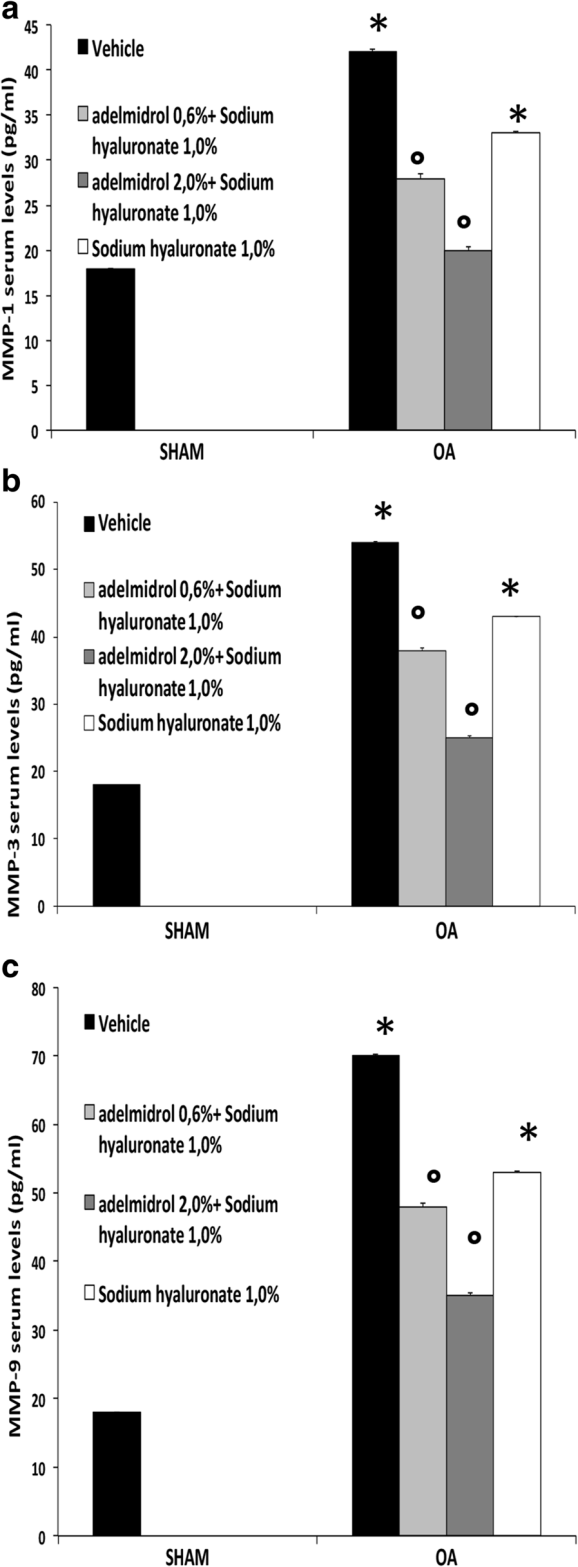
Effects of HA and adelmidrol combination on plasma levels of matrix metalloproteinases (*MMPs*). MMPs play a primary role in the downstream signaling pathways in osteoarthritis (*OA*) and cartilage degradation. The MIA + vehicle and 1% sodium hyaluronate group showed a markedly higher expression of MMP-1 (**a**), MMP-3 (**b**), and MMP-9 (**c**). Compared to the MIA group, the group treated with 0.6% adelmidrol + 1.0% sodium hyaluronate and, in particular, with 2% adelmidrol + 1.0% sodium hyaluronate showed a decrease in MMP-1, MMP-3, and MMP-9 expression (**a**–**c**, respectively). Data are means ± SEM of 10 rats for each group. *P < 0.05 versus sham, °*P* < 0.05 versus MIA + vehicle

The progressive destruction of articular cartilage is caused by a number of matrix-degrading enzymes produced by the chondrocytes and synovium. Among the various biomarkers associated with OA, MMPs play a primary role in the downstream signaling pathways in OA and cartilage degradation. The MIA + vehicle and 1% sodium hyaluronate group showed a markedly higher expression of MMP-1 (Fig. 4a), MMP-3 (Fig. 4b), and MMP-9 (Fig. 4c). However, compared to the MIA group, the group treated with 0.6% adelmidrol + 1.0% sodium hyaluronate and, in particular, 2% adelmidrol + 1.0% sodium hyaluronate showed a decrease in MMP-1, MMP-3, and MMP-9 expression (Fig. 4a, b and c, respectively).

### Effects of the HA and adelmidrol combination on OA articular cartilage degeneration

**Fig. 5:**
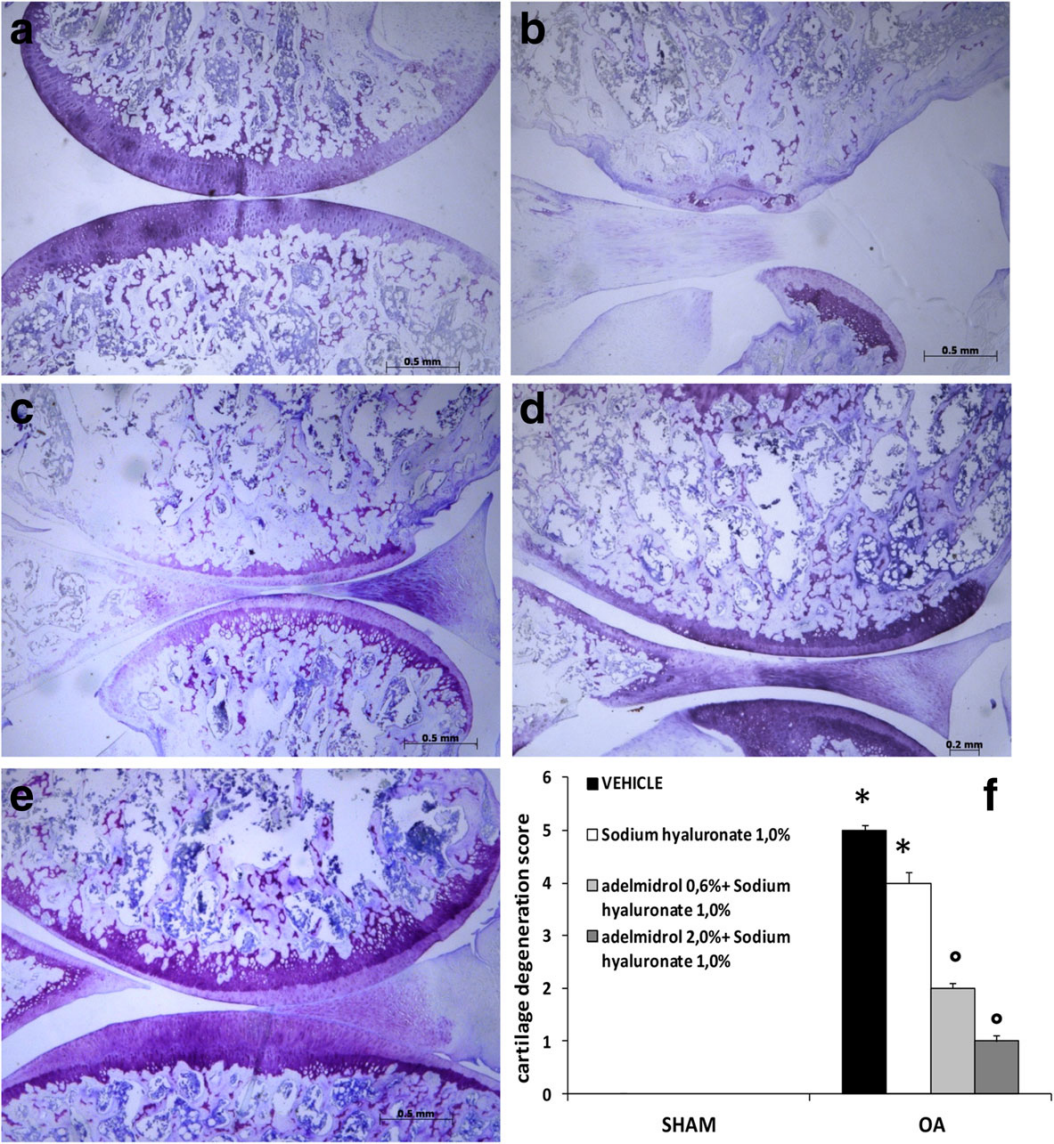
Effects of the combination of hyaluronic acid and adelmidrol on cartilage degeneration in osteoarthritis (*OA*) knee tissue. MIA-induced OA and tissue sample processing were carried out as detailed in the Methods section. No cartilage degeneration was observed in the sham group (**a**). Significant histopathological changes were evident in the MIA + solvent (**b**) and MIA + 1.0% sodium hyaluronate (**c**) groups, as indicated by surface irregularity, disorganization of articular cartilage with apparent cloning of chondrocytes in the transitional and radial zones, and an intact tidemark. The combination of HA and adelmidrol at the doses indicated (0.6% adelmidrol + 1.0% sodium hyaluronate (**d**), and 2% adelmidrol + 1.0% sodium hyaluronate (**e**)) significantly prevented damage to the cartilage structure, reduced cellular abnormalities, and prevented change of the tidemark induced by administration of MIA. Cartilage degeneration scoring was performed by an independent observer (**f**). Data are means ± SEM of 10 rats for each group. *P < 0.05 versus sham, °*P* < 0.05 versus MIA + vehicle

As indicated by toluidine blue O staining (Fig. 5), MIA intra-articular injection induced articular cartilage degeneration on the medial tibia platform in the MIA + solvent (Fig. 5b; cartilage degeneration scores in Fig. 5f) and MIA + 1.0% sodium hyaluronate (Fig. 5c) groups. Compared with knee joints in the MIA + solvent group, knees in the 0.6% adelmidrol + 1.0% sodium hyaluronate (Fig. 5d) group exhibited moderate cartilage degeneration; matrix and chondrocyte loss affected the middle to deep zone. The group with the higher treatment dose of 2% adelmidrol + 1.0% sodium hyaluronate showed no statistical improvement in cartilage degeneration (Fig. 5e). Sham knee joints (Fig. 5a) showed no obvious changes.

### Effects of the HA and adelmidrol combination on OA subchondral bone

**Fig. 6:**
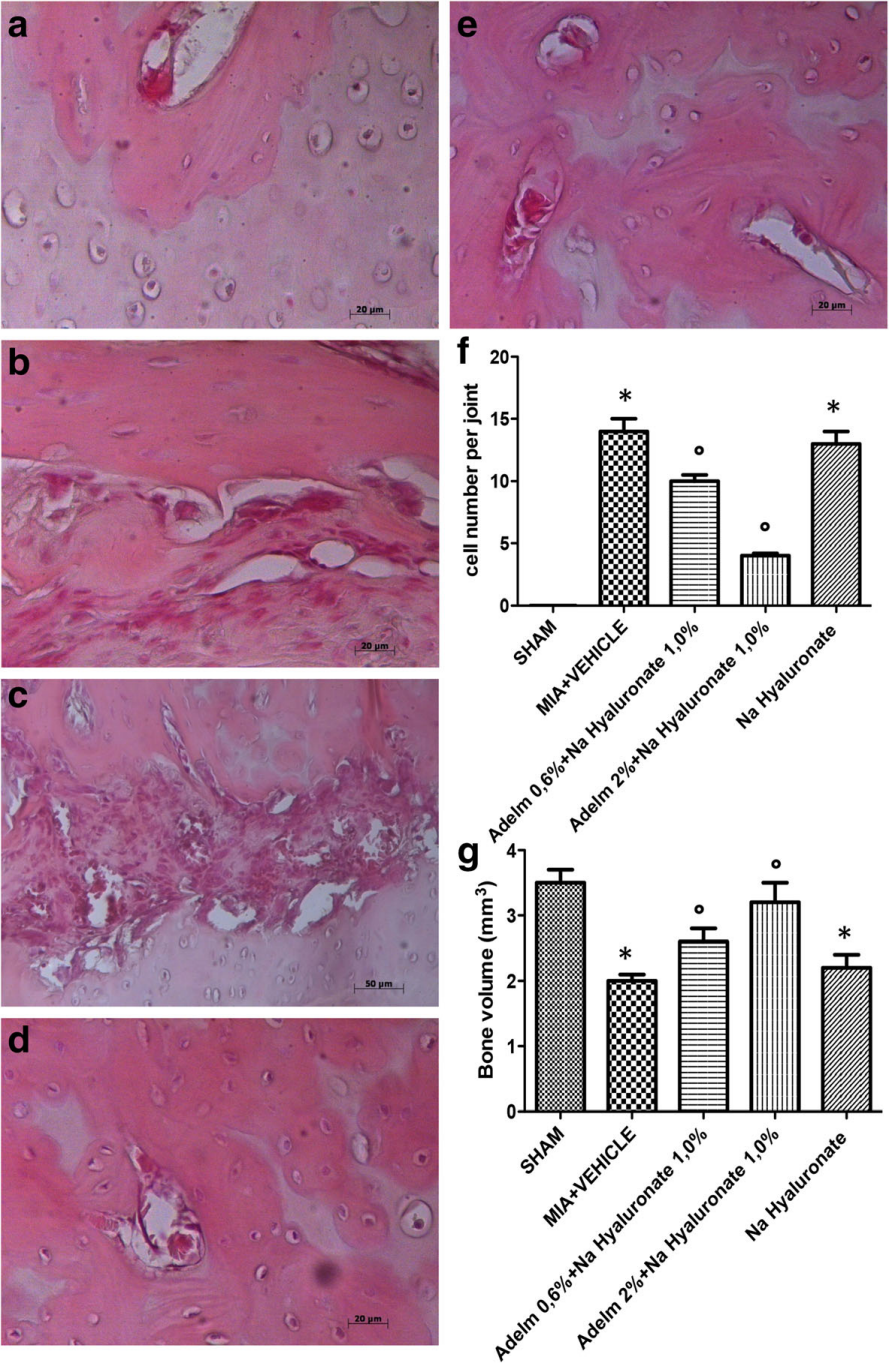
Effects of hyaluronic acid and adelmidrol combination on the pathogenesis of subchondral bone. In the monosodium iodoacetate (*MIA*) + solvent (**b**) and MIA + 1.0% sodium (*Na*) hyaluronate (**c**) knee groups, the number of osteoclasts was increased after MIA injection. Compared with knee joints in the MIA + solvent group, knees in the 0.6% adelmidrol (*Adelm*) + 1.0% sodium hyaluronate (**d**) group exhibited moderately increased numbers of osteoclasts. The higher treatment dose of 2% adelmidrol + 1.0% sodium hyaluronate showed no statistical improvement in cell numbers (**e**). There is no variation in cell numbers in sham knee joints (**a**). **f** Cell number per joint. **g** Moreover to quantify the structural changes in the subchondral bone in MIA-induced OA, each rat was scanned for bone volume density (mm^3^ ). Compared with the MIA + solvent and MIA + 1.0% sodium hyaluronate groups, 0.6% adelmidrol + 1.0% sodium hyaluronate and higher treatment dose of 2% adelmidrol + 1.0% sodium hyaluronate showed a marked increase in bone volume. Thus, treatment with 0.6% adelmidrol + 1.0% sodium hyaluronate and a higher treatment dose of 2% adelmidrol + 1.0% sodium hyaluronate showed significant and dose-dependent effects on MIA-induced deterioration of the subchondral bone. Data are means ± SEM of 10 rats for each group. *P < 0.05 versus sham, °*P* < 0.05 versus MIA + vehicle

In order to demonstrate the crucial role of subchondral bone in the pathogenesis and progression of OA, knee sections 21 days after MIA induction were examined for the number of osteoclasts and bone volume. In the MIA + solvent (Fig. 6b; cell number per joint in Fig. 6f) and MIA + 1.0% sodium hyaluronate (Fig. 6c) groups, the number of osteoclasts was increased after MIA injection. Compared with knee joints in the MIA + solvent group, knees in the 0.6% adelmidrol + 1.0% sodium hyaluronate (Fig. 6d) group exhibited a moderately increased number of osteoclasts. The higher treatment dose of 2% adelmidrol + 1.0% sodium hyaluronate showed no statistical improvement in cell numbers (Fig. 6e). There is no variation in cell numbers in Sham knee joints (Fig. 6a).

Moreover, to quantity the structural changes in the subchondral bone in MIA-induced OA, each rat was scanned for bone volume density (mm^3^) (Fig. 6g). Compared with the MIA + solvent and MIA + 1.0% sodium hyaluronate groups, 0.6% adelmidrol + 1.0% sodium hyaluronate and the higher treatment dose of 2% adelmidrol + 1.0% sodium hyaluronate showed a marked increase in bone volume (Fig. 6g). Thus, treatment with 0.6% adelmidrol + 1.0% sodium hyaluronate and the higher treatment dose of 2% adelmidrol + 1.0% sodium hyaluronate showed significant and dose-dependent effects on MIA-induced deterioration of the subchondral bone.

### Effects of the HA and adelmidrol combination on OA histopathology

**Fig. 7:**
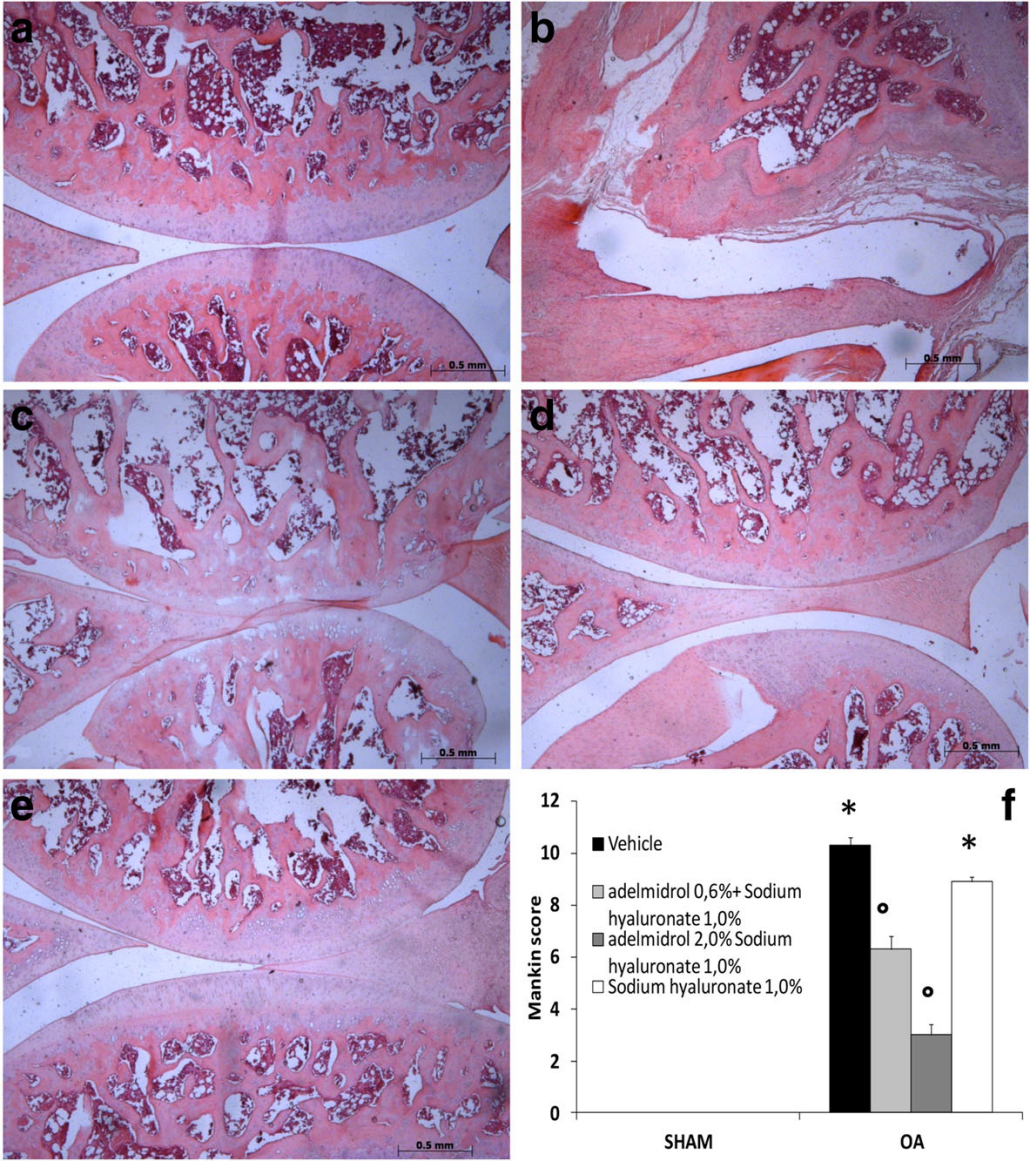
Effects of hyaluronic acid and adelmidrol combination on histological features of osteoarthritis (*OA*) knee tissue. MIA-induced OA and tissue sample processing were carried out as detailed in the Methods section. Knee sections from sham rats displayed normal architecture of the joint tissue (**a**; see Mankin Score in **f**). The MIA + solvent (**b**) and MIA + 1.0% sodium hyaluronate (**c**) groups showed surface layer fibrillation, a decrease in blood cells, multilayering in transition and radial zones, no pannus formation, and modified Mankin scores. The combination of HA and adelmidrol at the doses indicated (0.6% adelmidrol + 1.0% sodium hyaluronate (**d**), and 2% adelmidrol + 1.0% sodium hyaluronate (**e**)) reduced histological alterations induced by administration of MIA. Histological scoring was performed by an independent observer (**f**). Data are means ± SEM of 10 rats for each group. *P < 0.05 versus sham, °*P* < 0.05 versus MIA + vehicle

In order to demonstrate that adelmidrol promotes the beneficial and lubricant action of HA, knee sections were stained with hematoxylin and eosin 21 days after intra-articular injection of MIA. Histological examination by light microscopy showed irregularities and fibrillation in the surface layer, a decrease in blood cells and multilayering in transition and radial zones, no pannus formation, and modified Mankin scores (Fig. 7f) in the MIA + solvent and MIA + sodium hyaluronate 1.0% knees groups (Fig. 7b and c, respectively; see Mankin scores in Fig. 7f) compared to sham knees. The combination of HA and adelmidrol (0.6% adelmidrol + 1.0% solium hyaluronate (Fig. 7d) and 2% adelmidrol + 1.0% sodium hyaluronate (Fig. 7e)) reduced, in a dose-dependent manner, the histological alterations induced by administration of MIA.

## Discussion

OA is a complex disease with inflammatory mediators released by cartilage, bone, and synovium [27]. Numerous inflammatory mediators contribute to both degradative and nociceptive pathways associated with the progression of pathology. Inflammatory stimuli initiate a cascade of events, including the release of cytokines by chondrocytes, leading to complex biochemical and mechanical interplay with other biological mediators to induce OA and promote pain [27, 28].

Although the role of cytokines in the pathogenesis of OA is not yet clear, several in vitro studies support an elevated catabolic role for cytokines in the OA joint. IL-1β and TNF-α signaling, culminating in the activation of nuclear factor-κB and activator protein 1 transcription factors, can induce autocrine production of IL-1β and TNF-α as well as expression of other critical inflammatory and chrondrolytic mediators, including MMP1, MMP9, MMP13, nitric oxide, prostaglandin E2, IL-6, and pain [29].

Moreover it has been demonstrated that IL-1β induces an increase in levels of NGF [30]. NGF is a key factor in hyperalgesia associated with inflammation, whose protein is detected in OA synovial fluid [31]. NGF synthesis is highly correlated with the degree of cartilage degradation in human OA [32]. Clinical trials have demonstrated that acting against NGF leads to a dramatic reduction in OA pain [33]. In this study, we confirmed an increase in cytokine production and showed that treatment with HA in combination with adelmidrol in increasing doses significantly reduced pro-inflammatory cytokine levels and NGF expression.

The literature indicates that an important source of cytokines, specifically TNF-α and IL-1β, is also characterized by mast cell activation. There is a well-established correlation between mast cell numbers and total inflammatory infiltrate. Mast cells are potent regulators of vascular permeability and have a crucial role in the recruitment of leukocytes to OA joints. Degranulated mast cells have been found in OA synovium [34], and Buckley and Walls [35] reported a selective expansion and higher ratio of mast cell tryptase phenotype in OA synovium, a phenotype consistent with degranulation.

The present study confirms an increased infiltration of mast cells in the knee joint after MIA administration, and their consequent reduction after treatment with HA in combination with adelmidrol in increasing doses. Mast cells in the joint capsule become hyper-reactive due to joint inflammation, a process markedly influenced by the degradation of HA caused by the in situ release of β-hexosaminidase [36, 37]. In fact, OA animals and those treated only with HA displayed a significant degeneration of articular cartilage. Significant restoration of cartilage was achieved after treatment with increasing doses of HA and adelmidrol in combination.

Along with progressive loss of articular cartilage, OA is characterized by increased subchondral bone sclerosis with thickening of the cortical plate, extensive remodeling of the trabeculae, formation of new bone at the joint margins (osteophytes), and the development of subchondral bone cysts. Changes to subchondral bone are due to increased osteoclastic bone resorption and changes in bone density. Our study demonstrated that adelmidrol and hyaluronic acid treatment showed significant and dose-dependent effects on MIA-induced deterioration of the subchondral bone, reducing osteoclastic bone resorption.

Strong evidence associates subchondral bone alterations with cartilage damage and pain severity in OA [38]. In this study, motor functionality was evaluated by Walking Track Analysis. An SFI value of approximately zero indicates normal locomotor function, while a value close to –100 indicates significant impairment of locomotor function. As expected, intra-articular injection of MIA resulted in a significant increase in joint discomfort. Importantly, the combination of HA and adelmidrol, in a dose-dependent fashion, completely restored locomotor functionality.

## Conclusions

Our results clearly demonstrated that a combination of HA and adelmidrol dose-dependently produced a significant reduction in: 1) pain severity; 2) OA histopathology; 3) articular cartilage degeneration; 4) mast cell infiltration; 5) pro-inflammatory cytokines, MMPs, and NGF production; and 6) the degree of motor function.

The association of HA with adelmidrol is accompanied by a reduced depolymerization of HA, which would otherwise be promoted by intra-articular inflammation.

Moreover application of infiltrative adelmidrol produces a significant increase in endogenous PEA levels [39], which are found to be significantly reduced in joint inflammation [40]. Several of our works demonstrated the beneficial effects of PEA alone and in combination in different models of inflammation and pain [41, 42], such as in a mouse model of collagen-induced arthritis (CIA) [19]. Thus, local analgesic and anti-inflammatory effects observed with adelmidrol treatment could be useful in the treatment of inflammatory diseases associated with pain.

## Abbreviations

GC: Glucocorticoid
HA: Hyaluronic acid
IL: Interleukin
MIA: Monosodium iodoacetate
MMP: Matrix metalloproteinase
NGF: Nerve growth factor
NSAID: Nonsteroidal anti-inflammatory drug
OA: Osteoarthritis
PEA: Palmitoylethanolamide
PWL: Paw withdrawal latency
PWT: Paw withdrawal threshold
SFI: Sciatic nerve functionality index
TNF: Tumor necrosis factor
